# A case report of community-acquired *Pseudomonas aeruginosa* pneumonia complicated with MODS in a previously healthy patient and related literature review

**DOI:** 10.1186/s12879-019-3765-1

**Published:** 2019-02-08

**Authors:** Tao Wang, Yijun Hou, Ruilan Wang

**Affiliations:** Department of Critical Care Medicine, Shanghai General Hospital, Shanghai Jiao Tong University School of Medicine, Shanghai, Republic of China

**Keywords:** *Pseudomonas aeruginosa*, CAP, MODS

## Abstract

**Background:**

*Pseudomonas aeruginosa* is an unusual pathogen in community-acquired pneumonia, especially in previously healthy adults, but often indicates poor prognosis.

**Case presentation:**

We report a previously healthy patient who developed severe community-acquired pneumonia (CAP) caused by *P. aeruginosa*. He deteriorated to septic shock and multiple organ dysfunction syndrome (MODS) quickly, complicated by secondary hematogenous central nervous system (CNS) infection. After 1 month of organ support and antipseudomonal therapy, he had significant symptomatic improvement and was discharged from hospital. During treatment, the pathogen developed resistance to carbapenems quickly and the antibiotic regimen was adjusted accordingly.

**Conclusions:**

According to our case and related literature review, we conclude that more attention should be paid to community-acquired *Pseudomonas aeruginosa* pneumonia, because of its rapid progression and poor prognosis.

## Background

*Pseudomonas aeruginosa* is a gram-negative aerobic bacterium that can cause several types of infection including wound, urinary tract, and respiratory tract infections. The earliest description of *P. aeruginosa* pneumonia dates back to 1917 [1]. As one of the major pathogenic bacteria in hospital acquired pneumonia, it is usually seen in patients who have lung structural change, are immunocompromised, or have other specific risk factors. Although *P. aeruginosa* rarely causes pneumonia in healthy individuals, attention should be paid because of the high associated mortality and possibility of causing MODS.

We report a previously healthy patient who developed severe community-acquired pneumonia caused by *P. aeruginosa*, and provide a literature review of community-acquired *P. aeruginosa* pneumonia in terms of epidemiology, risk factors, pathogenesis and treatment.

## Case presentation

A 25-year-old patient with 2-day history of chest tightness and shortness of breath was admitted to the intensive care unit (ICU) due to worsening dyspnea over 6 h. Patient was previously healthy without history of contagious disease or sick contacts, and reported no tobacco or alcohol use. Patient was living in poor dwelling conditions.

Two days before admission, the patient started having sore throat and cough after flu-like symptoms that were self-treated with unknown medications without improvement. Six hours prior to admission, the patient developed worsening chest tightness, dyspnea, and difficulty talking while lying down. The patient was seen in an outside health care facility. There, the physical exam revealed the following: T 38.9 °C, BP 136/91 mmHg, HR 150 bpm, SpO2 99%with peripheral cyanosis and bilateral wheezing. Chest computed tomography (CT)showed an infection site in the right lung. The patient was given nebulized bronchodilators and intravenous corticosteroids without improvement and then transferred to the emergency department of our hospital.

**Fig. 1:**
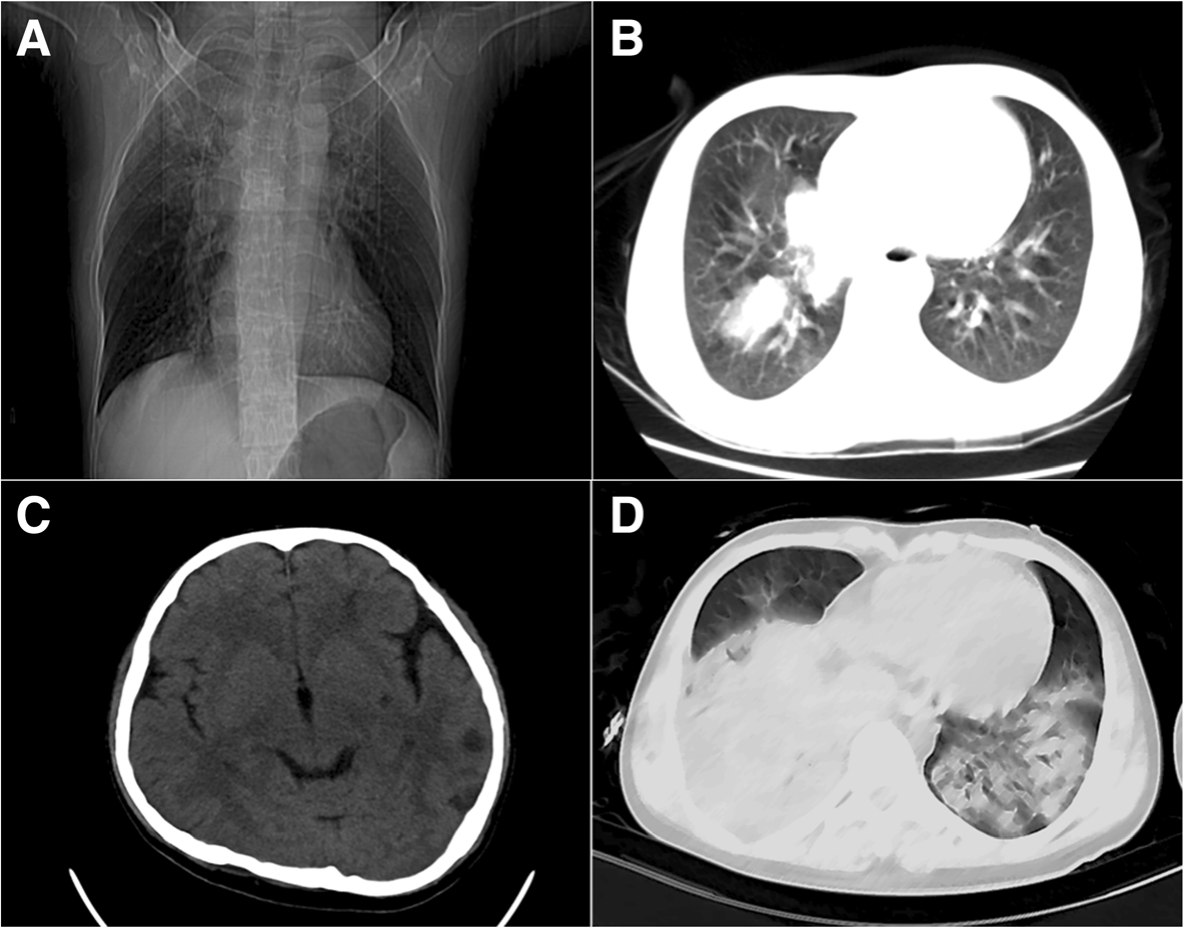
Chest CT on ICU admission (**a**, **b**: multiple nodular and patchy shadows in the right superior and inferior lobes); Repeat CT on Day 5 (**c**: scattered patches of low densities in the left hemisphere, **d**: multiple large consolidation areas, worse compared with previous imaging; suspicious multiple nodular shadows within the consolidation of right inferior lobe; mild pleural effusion in right lung)

Patient presented to emergency room (ER) with confusion, undetectable blood pressure, dyspnea, and chest retractions. Arterial blood gas (ABG) showed pH 7.35, PaCO2 18.00 mmHg, PaO2 83.00 mmHg, lactate 8.20 mmol/Land BE − 15.70 mmol/L. He was intubated immediately, complicated by difficult airway, laryngeal edema, and bloody sputum. Repeat CT showed multiple nodular and patchy shadows in right superior and inferior lobes (Fig. 1a, b). Head CT was normal. See Table 1 for Day 1 laboratory results. Oseltamivir and levofloxacin were given along with aerosol inhalation. Patient was then admitted to ICU.

**Table 1:** Laboratory results on Days 1 and 5

Laboratory test	Day1	Day5
White blood cell (*10^9^)/L)	0.39	20.65
Neutrophil percentage (%)	47.6	93%
Lymphocyte percentage (%)	46	3.8
Platelets (*10^9^/L)	150	27
Hemoglobin (g/L)	133	95
Prothrombin time (PT, s)	21.6	12.3
Activated partial thromboplastin time (APTT, s)	36	30.5
International normalized ratio (INR)	1.8	1.0
Potassium (mmol/L)	4.1	3.5
Sodium (mmol/L)	137	148
ALT/AST (U/L)	8/52	27/32
Total bilirubin (μmol/L)	22	17.6
Albumin (g/L)	30.5	32.6
Serum creatinine (μmol/L)	80	68.5
Blood urine nitrogen (BUN) (μmol/L)	5.79	5.28
Lactate (mmol/L)	8.2	5.2
Index of oxygenation (P/F ratio)	103	250
Procalcitonin (PCT)	100	11.0
BNP (p g/ml)	2590	>5000
Point-of-care ultrasound LVEF	30%	45–50%
Tests for influenza A and influenza B	NEGATIVE	

Physical exam on ICU admission was as follows: intubated and sedated, thin habitus, BP 131/67 mmHg, HR 156 bpm, RR 28/min, temperature 39.6 °C, SpO2 95%, mottled skin without petechiae, bilateral rough breath sounds without rales or rhonchi, bloody secretions aspirated from the ET tube, flat abdomen with mildly increased tension and absent bowel sounds. Neurologic examination revealed pupil size 2.0 mm bilaterally with obtunded light reflex, flexible neck without resistance, and normal muscle tension in all extremities. Diagnosis on admission: community-acquired pneumonia (severe), septic shock and MODS. See Table 1 for Day 1 lab results.

The patient was kept sedated and was given muscle relaxant for dyspnea relief. Physical cooling was used to improve temperature. Blood, urine, sputum samples were collected and sent for microbiology tests. We gave empiric treatment with linezolid and moxifloxacin (Day 1 to Day 2), together with oseltamivir (there was a flu epidemic in the local community) and intravenous immune globulin. Pulse-induced continuous cardiac output (PICCO) was administered for hemodynamic monitoring. Cardiac ultrasound showed that the left ventricular ejection fraction (LVEF) was 30% with significant elevation of brain natriuretic peptide (BNP). Viral myocarditis could not be ruled out considering previous symptoms of upper respiratory infection. We started fluid resuscitation with volume and rate adjusted based on hemodynamic monitoring. The patient had persistent hypotension and resistant hypoglycemia since admission and thus norepinephrine was started (Day 1 to Day 6) along with intravenous glucose.

On Day 2, there was no improvement of septic shock. Point-of-care ultrasound of chest showed large consolidation with moderate amount of pleural effusion in the right lung. Bronchoscopy showed mucosal hyperemia in main bronchi and the respective lobar, segmental bronchi. Large amounts of bloody secretions were seen in the right bronchi. Bronchoalveolar lavage(BAL) fluid and sputum smear showed gram-negative rods and we replaced moxifloxacin with meropenem. Continuous renal replacement therapy (CRRT) was started (Day 2 to Day 4) due to high lactate levels since admission (8.2 to 12.4 mmol/L). Repeat complete blood cell (CBC) showed continuous decrease in platelet counts (see Table 1) and linezolid was thus suspended. On Day 5, the result of BAL fluid culture indicated growth of *P. aeruginosa* (see Table 2). Based on drug-sensitivity test, levofloxacin was added to the regimen (levofloxacin and meropenem at this point).

**Table 2:** Culture results and drug sensitivity test results

Sample collection date	Day 1	Day	Day 2	Day 2	Day 6
Sample type	Blood	Sputum	BAL fluid	Throat swab	BAL fluid
Drug sensitivity results	*P. aeruginosa*	*P. aeruginosa*	*P. aeruginosa*	*P. aeruginosa*	*P. aeruginosa*
Amikacin	S sensitive<=16	S < =16	S < =16	S < =16	S < =16
Ciprofloxacin	S < =1	S < =1	S < =1	S < =1	S < =1
Gentamycin	S < =4	I intermediate 8	I 8	I 8	I 8
Levofloxacin	S < =2	S < =2	S < =2	S < =2	S < =2
Aztreonam	S < =4	S < =4	S < =4	S < =4	S < =4
Ceftazidime	S 4	S 4	S 4	S 4	S 4
Cefepime	S < =4	S 8	S 8	S 8	S < =4
Imipenem	S < =1	S 2	S 2	S 2	R resistant> 8
Meropenem	S < =1	S < =1	S < =1	S < =1	I 4
Piperacillin/tazobactam	S < =16	S < =16	S < =16	S < =16	S < =16
Tobramycin	S < =4	S < =4	S < =4	S < =4	S < =4

On Day 5, the patient had confusion after sedatives were withheld. Repeat head CT showed scattered patches of low densities in the left hemisphere (Fig. 1c). Chest CT showed multiple large consolidation areas (severe pneumonia), worse compared with previous imaging (Day 5); suspicious multiple nodular shadows within the consolidation of right inferior lobe; and mild pleural effusion in right lung (Fig. 1d), Second-generation sequencing of blood and BAL fluid showed quantities of *P. aeruginosa* duplication (samples collected on Day 5 and report available on Day 9). Lumbar puncture was done and cerebrospinal fluid (CSF)pressure was 23.5 cm H2O. CSF analysis showed elevated proteins. Due to positive blood culture of *P. aeruginosa*, we inferred that low densities were sites of hematogenous infection, with *P. aeruginosa* being the most likely pathogen, changed the antibiotic regimen to ceftazidime plus meropenem and performed a tracheotomy.

The patient had absent bowel sounds since admission and point-of-care ultrasound showed intestinal dilation without obvious peristalsis. Patient also developed severe diarrhea during treatment (stool volume approximately 1500 ml/day). Enema and gastric decompression were performed. *Clostridium difficile* infection could not be ruled out, and oral vancomycin was given along with probiotics. Later, the patient’s abdominal distension and diarrhea improved.

On Day 9, repeat BAL fluid culture was positive for *P. aeruginosa* and drug sensitivity test showed the development of carbapenem-resistance (previously carbapenem-sensitive). We changed the antibiotic regimen to ciprofloxacin plus ceftazidime. Patient’s temperature improved gradually and regained consciousness after sedatives were discontinued. Patient was weaned off ventilator on Day 10 and was given high-flow oxygen therapy. Brain magnetic resonance imaging (MRI) on Day 19 reported multiple foci of signal abnormalities in the left hemisphere, which were considered to be hematogenous brain abscesses. Repeat chest CT on Day 19 showed absorption of inflammation and encapsulated pleural effusion in the right lung. Repeat head CT reported no obvious lesions.

**Fig. 2:**
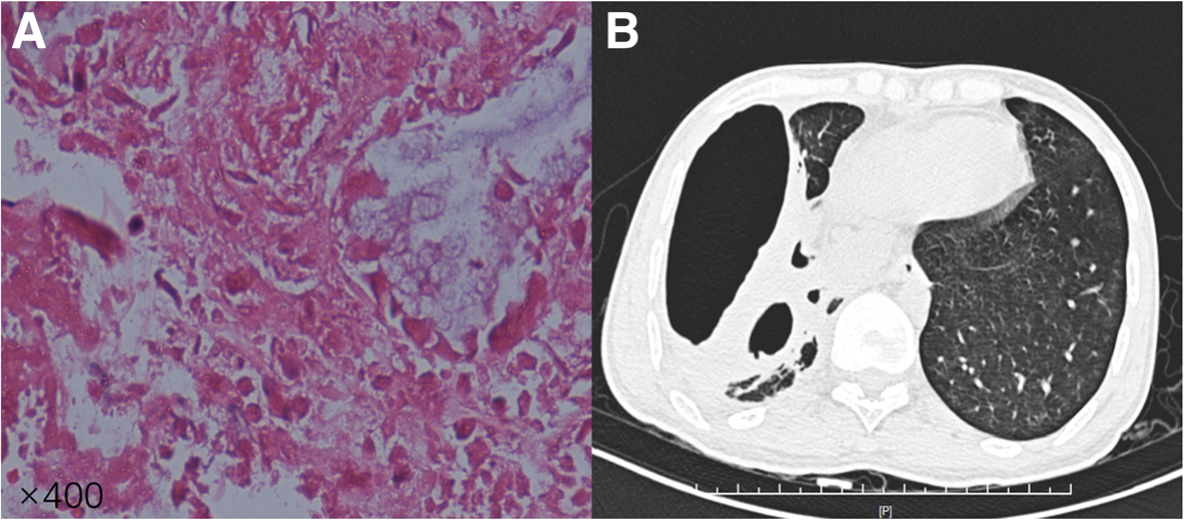
Microscopic exam of biopsy (**a**, × 400): hemorrhagic necrosis mixed with inflammatory exudates; chest CT before discharge (**b**):structural lung damage with remaining cavities

Since admission, patent had been coughing up thick sputum mixed with necrotic tissues, which repeatedly blocked the tracheotomy tube. Bronchoscopy revealed extensive necrotic tissue attaching to the walls of main bronchi. Biopsy showed hemorrhagic necrosis mixed with inflammatory exudates (Fig. 2a). Antibiotics were continued. After treatment and rehabilitation for a month, patient had significant symptomatic improvement and was discharged from hospital. Repeat chest CT before discharge showed structural lung damage with remaining cavities. (Fig. 2b).

## Discussion and conclusions

This patient was diagnosed with severe community-acquired pneumonia (CAP) due to *P. aeruginosa*, which progressed to septic shock quickly. It was complicated by secondary hematogenous CNS infection and MODS involving cardiovascular, hematologic, central nervous, and gastrointestinal systems. During treatment, the pathogen developed resistance to carbapenems quickly and the antibiotic regimen was adjusted accordingly. The patient ended up with symptomatic improvement and was discharged from hospital, though unilateral structural lung damage and multiple cavities remained.

In CAP, *P. aeruginosa* is rarely identified as the pathogenic agent, accounting for only 0.4–6.9% in reported cases of CAP requiring hospitalization [2, 3] and 1.8–8.3% in CAP requiring ICU admission [4–8]. It is very rare in previously healthy patients since *P. aeruginosa* usually causes infections in patients who have lung structural change, are immunocompromised, or have other specific risk factors. In the English-language literature reported from 1966 to 2000, Todd F [9] et al. identified only 11 publications describing a total of 13 cases.

Most *P. aeruginosa*-caused CAP is seen in patients with structural lung diseases, chronic obstructive pulmonary disease (COPD) or cystic fibrosis. Therefore, the 2007 American Thoracic Society (ATS) / Infectious Diseases Society of America (IDSA) guidelines recommended empirical treatment against *P. aeruginosa* in community-acquired pneumonia (CAP) patients with the following specific risk factors: 1) structural lung disease, like bronchi, 2) recurrent exacerbations of COPD requiring corticosteroid/antibiotic treatment, 3) antibiotic use before admission,4) immunocompromised status. However, by analyzing the data of 402 cases, ORIOL SIBILA et al. [10] found that current risk factors for CAP due to *P. aeruginosa* in the CAP guidelines identified only one-third of the patients admitted with CAP due to *P. aeruginosa*, with the other two-thirds undetected. They also found that chronic heart failure, cerebrovascular disease, advanced age and smoking were risk factors for CAP due to *P. aeruginosa*. Catia Cillóniz et al. [2, 11] found that malnutrition was another important risk factor for *P. aeruginosa*-caused CAP. Several studies [12, 13] also found that fatal *P. aeruginosa* pneumonia in previously healthy patients was associated with contaminated hot tubs. Influenza may be a risk factor for *P. aeruginosa* infection, there are some reports [14, 15] of *P. aeruginosa* co-infection with influenza A (H1N1). Influenza viral infection contributes to respiratory epithelial cell dysfunction and death through disruption of protein synthesis and induction of apoptosis, allowing for increased bacterial adherence and invasion [16].

Compared to pneumonia caused by other pathogens, *P. aeruginosa* CAP exhibits rapid progression, high severity and poor prognosis. *P. aeruginosa* CAP usually has a higher CURB-65 score and pneumonia severity index (PSI) than pneumonia caused by other pathogens, with mortality approximately 18–61% [17, 18]. In severe *P. aeruginosa* CAP, mortality of those who developed progressive septic shock and MODS can reach as high as 50–100% [2]. In death cases, Todd F [9] reported a median time of 11 h from admission to death. Advanced age(>65y), chronic liver disease, acute renal failure, requirement of ICU admission, and improper initial antibiotic use might be risk factors for poor prognosis. Even in survival cases, the foci of infection usually develop into fibrous scar tissue, or repeated infections requiring long-term antibiotic treatment. In our case, the patient developed necrotic pneumonia with cavity formation.

The progression and prognosis of such cases might be associated with various pathogeneses of *P. aeruginosa* [19, 20] :1)*P. aeruginosa* secretes toxins into the extracellular environment and into host cells. For example, through the type III secretion system (TTSS), *P. aeruginosa* injects toxins (e.g, ExoS, ExoT, ExoU) that change host cell activities and disrupt host cell actin cytoskeletons, block phagocytosis, and cause cell death; 2) Bacterial surface factors such as flagella, pili and lipopolysaccharide induce host inflammatory responses; 3) QS (quorum sensing), functioning as the connection between neighboring bacteria, plays a role in the regulation of a wide variety of processes including biofilm formation and production of numerous toxins; 4) *P. aeruginosa* secretes various enzymes and cytotoxins, such as elastase, alkaline protease and exotoxin A, that either disrupt the integrity of the epithelial barrier by disrupting epithelial cell tight junctions or cause direct tissue damage and necrosis.

The patient in this case developed rapid antimicrobial resistance to carbapenems. Despite a number of studies on antimicrobial resistance of hospital-acquired *P. aeruginosa* infection, the number of studies on resistance of community-acquired *P. aeruginosa* is limited. After analyzing drug sensitivity reports of 77 *P. aeruginosa* CAP cases, researchers found 32% were multi-drug resistant strains and 68% were sensitive strains [2]. During treatment, these bacteria acquire antimicrobial resistance by several mechanisms, including reduced permeability, enzymatic degradation, and active efflux [21]. Development of resistance is also associated with gene mutations. For example, repressed oprD expression leads to carbapenem resistance; mexX mutation causes resistance to aminoglycosides and fluoroquinolones [22]. Mutations of gyrA and gyrB(gyrase) also result in resistance to fluoroquinolones [23]. In addition, biofilm formation also contributes to antimicrobial resistance in *P. aeruginosa*. Biofilms are bacterial cities, highly organized, microbial communities encased in a polysaccharide matrix and attached to the surfaces of implants or airways. These multi-drug resistant variants in these colonies contribute to the high resistance of biofilms to antimicrobials.

Regarding treatment of *P. aeruginosa* CAP, guidelines recommend empirical treatment for these who have risk factors for *P. aeruginosa* infection. Early administration of proper antibiotics may improve the outcomes for such patients [24]. For patients with suspected *P. aeruginosa*-caused severe pneumonia, combination antibiotic therapy should be administered within an hour [25]. For critically ill patients admitted to the ICU, guidelines recommend use of an antipseudomonal β-lactam (piperacillin-tazobactam, cefepime, imipenem, or meropenem) plus an antipseudomonal fluoroquinolone; or the above β-lactam plus an aminoglycoside and azithromycin; or the aboveβ-lactam plus an aminoglycoside and a fluoroquinolone [26]. Once *P. aeruginosa* is confirmed to be the pathogenic agent, antibiotic regimen should be adjusted to be more targeted. Targeted therapy recommended by guidelines includes an antipseudomonal β-lactam plus an aminoglycoside or a fluoroquinolone, with the alternative being an aminoglycoside plus a fluoroquinolone. According to a study by Cillóniz [2], inappropriate therapy occurred in 64 and 77% cases of *P. aeruginosa* CAP and multi-drug resistant *P. aeruginosa* CAP, respectively. In light of the high severity and rapid progression, patients with such conditions should be monitored closely and should receive frequent organ function evaluations.

According to our case and related literature review, we conclude that more attention should be paid to community-acquired *Pseudomonas aeruginosa* pneumonia because of its rapid progression and poor prognosis.

## Abbreviations

ATS: American Thoracic Society
BAL: Bronchoalveolar lavage
BNP: Brain natriuretic peptide
CAP: Community-acquired pneumonia
CNS: Central nervous system
COPD: Chronic obstructive pulmonary disease
CRRT: Continuous renal replacement therapy
CSF: Cerebrospinal fluid
CT: Computed tomography
ER: Emergency room
ICU: Intensive care unit
IDSA: Infectious Diseases Society of America
LVEF: Left ventricular ejection fraction
MODS: Multiple organ dysfunction syndrome
PICCO: Pulse -induced continuous cardiac output
